# A Unique Case of Biventricular Arrhythmogenic Cardiomyopathy

**DOI:** 10.7759/cureus.81752

**Published:** 2025-04-05

**Authors:** Aakash Rana, Jack Xu, Jin Zhao

**Affiliations:** 1 Medicine, Central Arkansas Veterans Healthcare System, Little Rock, USA; 2 Cardiology, Novant Health, Winston-Salem, USA

**Keywords:** arrhythmogenic cardiomyopathy, arrhythmogenic disorders, arrhythmogenic right ventricular cardiomyopathy, epsilon waves, sudden cardiac death

## Abstract

Arrhythmogenic cardiomyopathy is a type of heart disease that is a well-recognized cause of sudden cardiac death among the young population. It can affect the right ventricle, left ventricle, or both ventricles of the heart. This condition involves the replacement of heart muscle with fatty tissue, which can disrupt the heart’s normal electrical and mechanical function, leading to arrhythmias, heart failure, and increased risk of sudden cardiac death. We report the case of a 62-year-old man who came to the emergency room with nausea, vomiting, and palpitations. After further evaluation, he was diagnosed with heart failure secondary to biventricular arrhythmogenic cardiomyopathy. The patient was found to have a genetic mutation in the RYR2 gene, which usually causes dilation of the right ventricle. However, in this case, both the right and left ventricles were dilated, which is unusual since RYR2 mutations are typically linked to right ventricle dilation only. This may represent a potentially novel phenotypic manifestation of the disease associated with this mutation, as no other cases have been reported in the literature.

## Introduction

Arrhythmogenic cardiomyopathy (ACM) is a relatively under-recognized hereditary cardiomyopathy that affects the right ventricle (RV), left ventricle (LV), or both. It is characterized pathologically by fibrofatty replacement of the myocardium [1]. The common presentation of this disease primarily involves the right ventricle (RV) and was originally called arrhythmogenic right ventricular cardiomyopathy (ARVC). However, recent research has uncovered other diseases involving the left ventricle (LV) [2]. The current classification of ACM encompasses the following main phenotypes: (1) the original ARVC phenotype, which is marked by predominant RV involvement without LV abnormalities (referred to as the ‘dominant-right’ variant); (2) the phenotypic variant characterized by equal involvement of both ventricles (‘biventricular’); and (3) the variant featuring predominant LV involvement with little to no RV abnormalities (‘dominant-left’), also known as arrhythmogenic left ventricular cardiomyopathy (ALVC) [3,4]. ACM is typically inherited in an autosomal dominant pattern with variable penetrance and incomplete expression [5,6]. Prior studies have shown that around two-thirds of patients affected with ACM have a positive genetic test. Based on current research, eight known genes are responsible for most of the pathogenesis of ARVC [7]. Among these genes, five are desmosomal genes (PKP2, DSP, DSC2, DSG2, and JUP), and three are non-desmosomal genes (TMEM43, DES, and PLN) [7]. Cardiac desmosomes are specialized cell-to-cell adhesion junctions that provide strong mechanical links between cardiomyocytes, crucial for coordinated heart function and preventing cell separation during contraction. Studies have demonstrated that 50% to 60% of patients with ARVC have gene mutations encoding for desmosomal proteins [7]. PKP2 is the most frequently reported gene mutation, accounting for 20% to 46% of clinically manifest ARVC cases [7]. Studies indicate that the most frequent gene deficiencies causing ALVC are mutations in the DSP, phospholamban (PLN), and filamin C (FLNC) genes [8].

Most patient presents with symptoms of heart palpitations, syncope, or even sudden death. ACM is recognized as a leading cause of sudden cardiac death (SCD) in young adults ≤ 35 years of age and may account for up to 10% of cardiovascular deaths in the < 65 age group [9]. ACM is diagnosed using a scoring system with major and minor criteria, as outlined in the 2023 European Task Force guidelines, instead of a single test [10-11]. The management of ACM involves clinical management, lifestyle changes, usage of beta-blockers and anti-arrhythmic drugs, catheter ablation, and implantable cardioverter-defibrillator (ICD) placement [1].

We present a case report of a 62-year-old man who presented with nausea/vomiting and palpations in the emergency room with further work-up for heart failure concerning biventricular arrhythmogenic cardiomyopathy.

## Case presentation

**Figure 1:**
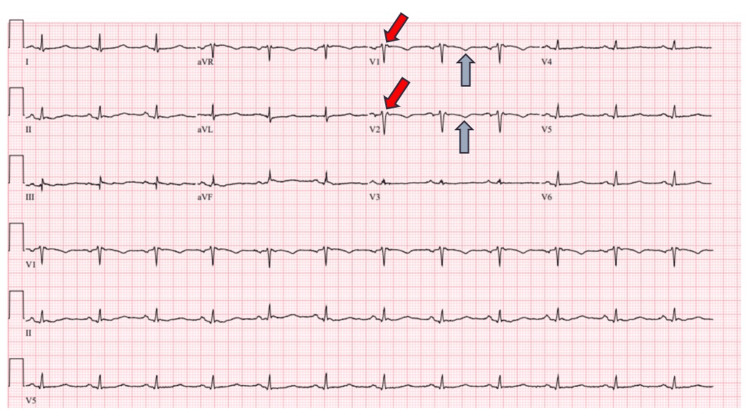
Electrocardiogram showing epsilon waves (red arrow) and T-wave inversions (grey arrows) in V1 and V2.

A 62-year-old male presented to the emergency room with nausea/vomiting and palpitations. The patient was found to be in supraventricular tachycardia with heart rates in the 220s on electrocardiogram concerning for atrioventricular nodal reentrant tachycardia/atrioventricular reentrant tachycardia (AVNRT/AVRT), with no response to adenosine. The patient was started on a diltiazem drip for rate control. A repeat ECG showed atypical atrial flutter with 2:1 block. Transthoracic echocardiogram (TTE) showed a decreased left ventricular ejection fraction (LVEF) of 20-25%, enlarged right ventricle, and right atrium. Left heart catheterization showed non-obstructive coronary artery disease. Right heart catheterization showed right atrium pressure of 13 mmHg, right ventricle systolic/diastolic pressure of 26/11 mmHg, and normal pulmonary capillary wedge pressure, but could not measure pulmonary artery pressure due to technical difficulties. He underwent a transesophageal echocardiogram (TEE) with direct cardioversion on this admission and was successfully converted back to normal sinus rhythm. No shunt was noticed on TEE. The patient was discharged from the hospital on goal-directed medical therapy for heart failure. The patient was seen in the heart failure clinic in eight weeks. The ECG from the clinic visit showed precordial T-wave inversion and epsilon waves in V1-V2 (Figure 1).

TTE from the clinic visit showed improvement in LVEF to 35-40% from 20-25% with dilated right ventricle and right atrium (Video 1).

**Video 1:** Transthoracic echocardiography (TTE) in four-chamber view showing dilation of the right atrium, right ventricle with left ventricular ejection fraction 35-40%.

**Figure 2:**
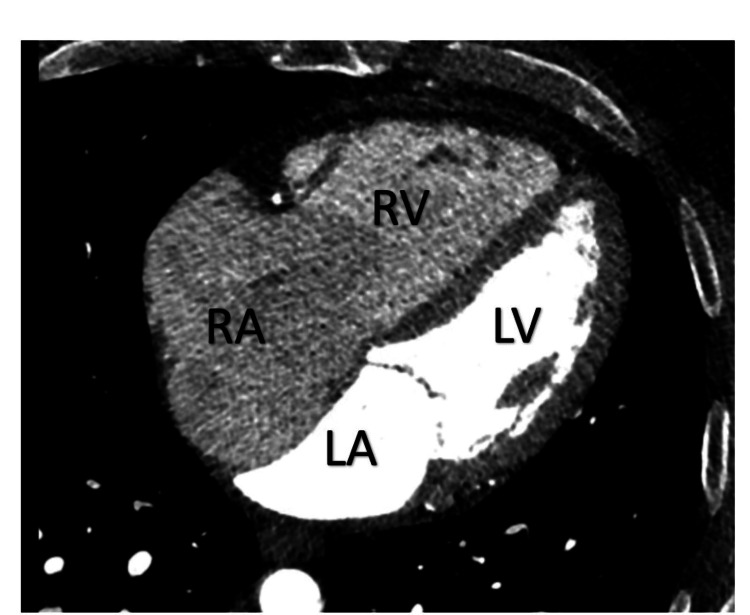
Computed tomography (CT) of heart in four-chamber view shows marked enlargement of the RA and RV. RA: right atrium, RV: right ventricle, LA: left atrium, LV: left ventricle.

Computed tomography (CT) of the heart was obtained for further evaluation that showed no evidence of intracardiac/extracardiac shunt that could explain the right-sided chamber enlargement, including the right atrium, right ventricle, coronary sinus, superior vena cava, and inferior vena cava (Figure 2).

**Figure 3:**
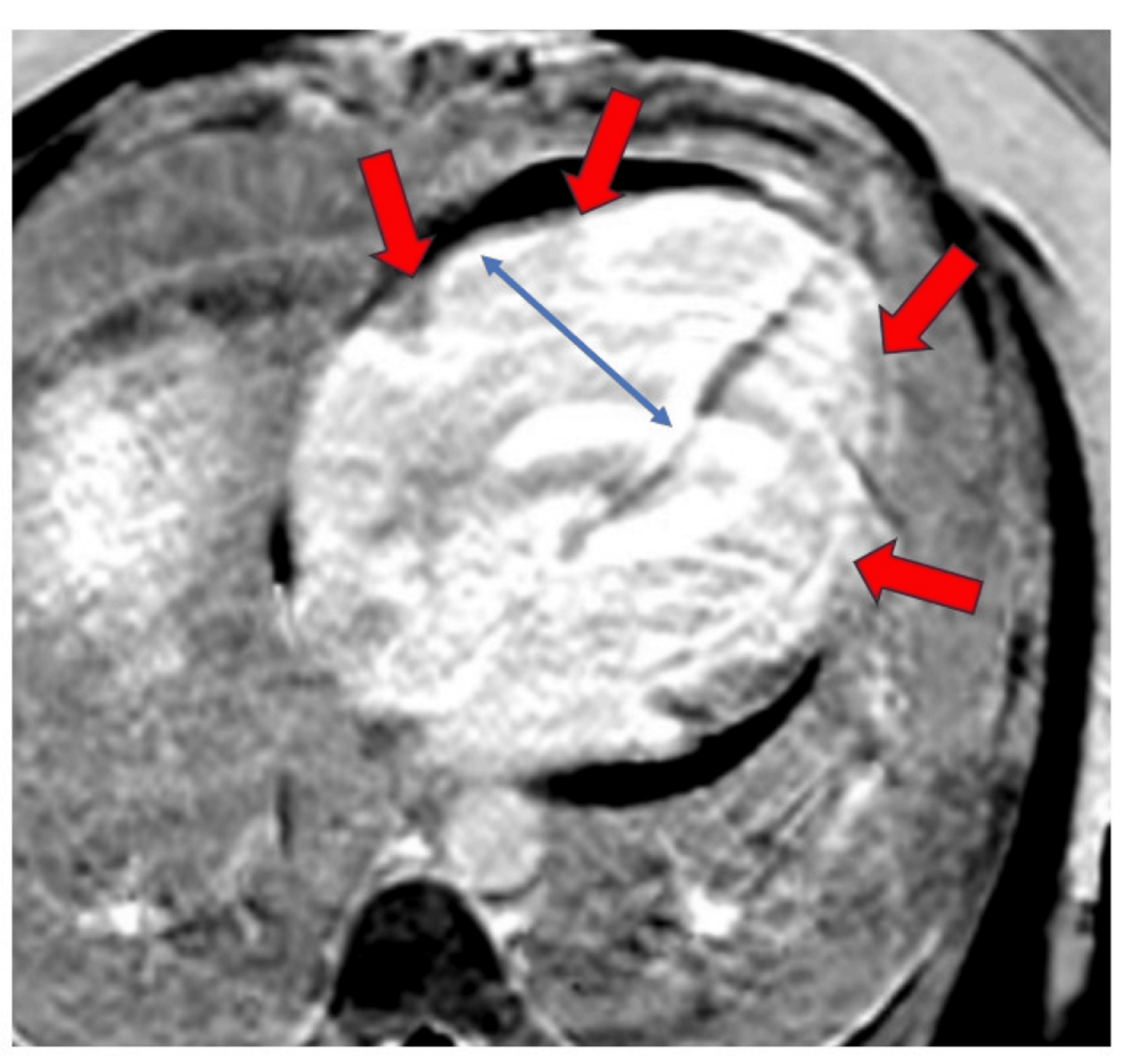
Cardiac MRI reveals late gadolinium enhancement in both the right and left ventricles (red arrow), along with dilation of the right ventricle (blue double-sided arrow) and “D-septum,” concerning for septal flattening in diastole, suggestive of right ventricular pressure overload.

Cardiac magnetic resonance imaging (MRI) was obtained to evaluate the etiology of cardiomyopathy. Cardiac MRI showed late gadolinium enhancement of the right ventricle, left ventricle, dilation of the right ventricle, and “D-septum,” concerning for septal flattening in diastole, suggestive of right ventricular pressure overload (Figure 3).

No apparent extracardiac shunt was noticed with a right ventricular ejection fraction of 28% and left ventricular ejection fraction of 44%. The above cardiac MRI findings in the setting of ECG findings were concerning for biventricular ACM. A 28-day event monitor was obtained for evaluation of arrhythmia in the setting of ACM, which showed multiple runs of non-sustained ventricular tachycardia. The patient had an RYR2 gene testing that was classified as a pathological mutation. No prior family history of ACM was noted. Genetic testing was also recommended for his family. Due to severe right ventricular dysfunction in a male patient with LVEF below 50% and a pathological RYR2 gene mutation, the patient met Class IIb recommendation for ICD placement for prevention of sudden cardiac death. Follow-up device interrogation has shown no therapies.

## Discussion

Diagnosing ACM is difficult and relies on a qualitative scoring system with major and minor criteria, as outlined in the 2023 European Task Force guidelines, instead of a single standard test, as mentioned in Table 1 [10-11].

**Table 1:** The 2023 European Task Force diagnostic criteria for arrhythmogenic ventricular cardiomyopathy. ACM, arrhythmogenic cardiomyopathy; BSA, body surface area; ECG, electrocardiogram; EDV, end diastolic volume; EF, ejection fraction; LBBB, left bundle branch block; LGE, late gadolinium enhancement; LV, left ventricle; RBBB, right bundle branch block; RV, right ventricle; RVOT, right ventricular outflow tract. Adapted from [11]. Open access article, distributed under the terms of the Creative Commons CC-BY license, which permits unrestricted use, distribution, and reproduction in any medium, provided the original work is properly cited.

Category	Criteria for RV involvement	Criteria for LV involvement
1. Morpho-functional ventricular abnormalities	Major	Minor
	• Regional RV akinesia, dyskinesia, or aneurysm plus one of the following:	• Global LV systolic dysfunction, with or without LV dilatation (increase of LV EDV according to the imaging test-specific nomograms for age, sex, and BSA)
	- global RV dilatation (increase of RV EDV according to the imaging test specific nomograms for age, sex and BSA)	
	or	
	- global RV systolic dysfunction (reduction of RV EF according to the imaging test-specific nomograms for age and sex)	
	Minor	
	• Regional RV akinesia, dyskinesia, or aneurysm of RV free wall	
2. Structural myocardial abnormalities	Major	Major
	• Fibrous replacement of the myocardium in ≥≥1 sample, with or without fatty tissue, at histology	• “Ring-like” LV LGE (subepicardial or midmyocardial stria pattern) of ≥≥3 segments (confirmed in 2 orthogonal views)
	Minor	Minor
	• Unequivocal RV LGE (confirmed in 2 orthogonal views) in ≥≥1 RV region(s) (excluding tricuspid valve)	• LV LGE (subepicardial or midmyocardial stria pattern) of 1 or 2 Bull’s Eye segment(s) (in 2 orthogonal views) of the free wall, septum, or both (excluding patchy, focal, or septal junctional LGE)
3. ECG repolarization abnormalities	Major	Minor
	• Negative T waves in right precordial leads (V1, V2, and V3) or beyond in individuals ≥≥14 years old (in the absence of complete RBBB and not preceded by J-point/ST-segment elevation)	• Negative T waves in left precordial leads (V4–V6) (in the absence of complete LBBB)
	Minor	
	• Negative T waves in leads V1 and V2 in males ≥≥14 years old (in the absence of RBBB and not preceded by J-point/ST-segment elevation)	
	• Negative T waves beyond V3 in the presence of complete RBBB	
	• Negative T waves beyond V3 in individuals <<14 years old	
4. ECG depolarization and conduction abnormalities	Minor	Major
	• Epsilon wave (reproducible low-amplitude signals between the end of QRS complex to the onset of the T wave) in the right precordial leads (V1 to V3)	• Low QRS voltages (<<0.5 mV peak to peak) in all limbs leads in the absence of other causes (e.g., cardiac amyloidosis, obesity, emphysema, or pericardial effusion)
	• Terminal activation duration of QRS ≥≥55 ms measured from the nadir of the S wave to the end of the QRS, including R’, in V1, V2, or V3 (in the absence of complete RBBB)	
5. Arrhythmias	Major	Minor
	• Frequent ventricular extrasystoles (>>500 per 24 h), non-sustained or sustained ventricular tachycardia of LBBB morphology with non-inferior axis	• Frequent (>>500 per 24 h) or exercise-induced ventricular extrasystoles with a RBBB morphology or multiple RBBB morphologies (excluding the “fascicular pattern”)
	Minor	• Non-sustained or sustained ventricular tachycardia with a RBBB morphology (excluding the “fascicular pattern”)
	• Frequent ventricular extrasystoles (>>500 per 24 h), non-sustained or sustained ventricular tachycardia of LBBB morphology with inferior axis (“RVOT pattern”)	• History of cardiac arrest due to ventricular fibrillation or sustained ventricular tachycardia of unknown morphology
	• History of cardiac arrest due to ventricular fibrillation or sustained ventricular tachycardia of unknown morphology	
6. Family history/genetics	Major	
	• Identification of a pathogenic ACM-gene variant in the patient under evaluation	
	• ACM confirmed in a first-degree relative who meets the diagnostic criteria	
	• ACM confirmed pathologically at autopsy or surgery in a first-degree relative	
	Minor	
	• Identification of a likely pathogenic ACM-gene variant in the patient under evaluation	
	• History of ACM in a first-degree relative in whom it is not possible or practical to determine whether the family member meets diagnostic criteria	
	• Premature sudden death (<<35 years of age) due to suspected ACM in a first-degree relative	
	• ACM confirmed pathologically or by diagnostic criteria in a second-degree relative	

The hallmark clinical features of ACM are mainly associated with ventricular arrhythmias and sudden cardiac death (SCD). The natural progression of ACM is traditionally described in four stages: (1) the concealed phase, during which the patient is symptom-free or only mildly symptomatic, but has structural changes noticed via echocardiography (This phase can still present with sudden cardiac death or aborted SCD); (2) the overt phase, where the patient experiences more clear symptoms such as arrhythmias, palpitations, and more noticeable structural changes; (3) end-stage disease, characterized by right ventricular dilation and dysfunction; and (4) biventricular failure, resembling dilated cardiomyopathy [12].

**Figure 4:**
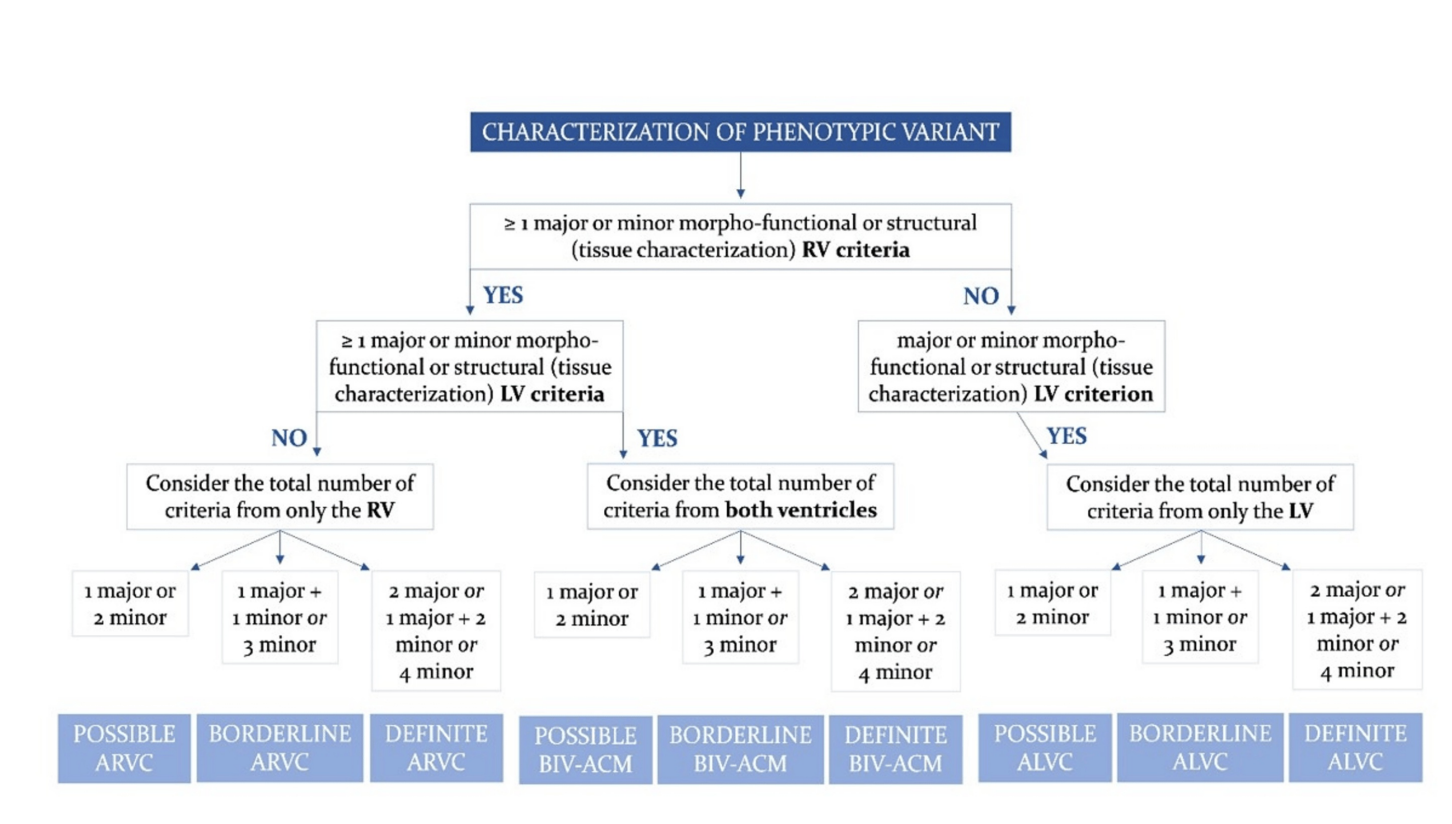
Flowchart for phenotypic characterization of ACM. ACM, arrhythmogenic cardiomyopathy; ALVC, arrhythmogenic left ventricular cardiomyopathy; ARVC, arrhythmogenic right ventricular cardiomyopathy; BIV-ACM, biventricular arrhythmogenic cardiomyopathy; LV, left ventricle; RV, right ventricle. Adapted from [11]. Open access article, distributed under the terms of the Creative Commons CC-BY license, which permits unrestricted use, distribution, and reproduction in any medium, provided the original work is properly cited.

The 2023 European Task Force diagnostic criteria involve three consequent steps in the ACM diagnostic process: (1) ventricular involvement; (2) phenotypic definition; and (3) etiology and classification involving Table 1 and Figure 4 to establish a precise diagnosis of ACM with its phenotypic variant [10-11]. The first step, based on dominant ventricular involvement, allows identification of three primary phenotypic variants: (1) arrhythmogenic right ventricular cardiomyopathy (ARVC), (2) arrhythmogenic biventricular cardiomyopathy, and (3) arrhythmogenic left ventricular cardiomyopathy (ALVC), characterized by isolated LV abnormalities. The second stage of the diagnostic process focuses on identifying the ACM phenotype by assessing the fulfillment of criteria related to both RV and LV involvement. According to the 2023 European Task Force criteria, a diagnosis of ACM requires meeting at least one criterion, either major or minor, from either the first category (morpho-functional abnormalities) or the second category (structural abnormalities) [10-11]. This distinction is pivotal as ACM is fundamentally a structural heart disease. The third stage in the diagnostic process involves identifying the etiology and classification with further work-up with genetic testing [10-11].

In our case, the patient presented to the emergency room with atrial flutter rather than ventricular tachycardia with LBBB, typically seen in typical ACM presentation. The patient was diagnosed with new-onset heart failure during his hospitalization and was found to show a decreased LVEF of 20-25%, enlarged right ventricle, and right atrium. To investigate this morphology further, cardiac MRI was performed and revealed late gadolinium enhancement in both the right and left ventricles, along with dilation of the right ventricle and the “D-septum”. The ECG showed T-wave inversion in leads V1 and V2, along with epsilon waves in those leads. The patient satisfied two major criteria for RV involvement, one related to morpho-functional ventricular abnormalities and the other to structural myocardial abnormalities, along with two minor criteria each in ECG repolarization abnormalities and ECG depolarization and conduction abnormalities. The patient met two minor LV involvement criteria: morpho-functional ventricular and structural myocardial abnormalities. Based on Figure 4, with the above major and minor criteria, our patient has a definite biventricular ACM. Based on the imaging findings, he was classified in the end-stage disease phase due to right ventricular dilation, despite reporting no symptoms at the time of diagnosis. Our patient had a comprehensive heart failure gene panel performed with RYR2 gene classification as a pathological mutation. The RYR2 gene, which encodes the cardiac ryanodine receptor, plays an essential role in excitation and contraction coupling in cardiac myocytes [13]. RYR2 mutations disrupt the normal function of the RyR2 channel, leading to excessive calcium release from the sarcoplasmic reticulum (SR) during diastole, which can trigger arrhythmias. Per literature review, RYR2 missense pathogenic mutations are associated with conventional ARVC involving RV dilation and wall motion abnormalities without mutation in desmosome genes [13]. In our case, we have RV dilation and LV dilation, which makes it unique since RYR2 missense mutations are typically associated with RV dilation. This could represent a potentially novel phenotypic presentation of the disease since no other case has been presented in the literature.

The management of ACM involves clinical management, pharmacological options, catheter ablation, and placement of ICD. Clinical management involves lifestyle modifications advised for all ACM patients due to the strong link between endurance exercise and the increased risk of ventricular arrhythmias. In addition to avoiding competitive sports, patients with ARVC should avoid any physical activity that triggers palpitations or pre-syncope [14-15]. Pharmacologic therapies involve using antiarrhythmic agents such as amiodarone and sotalol, cardio-selective β-blockers such metoprolol XL, and heart failure drug therapy [16]. Catheter ablation is recommended in ACM patients with incessant ventricular tachycardia (VT) despite maximal pharmacological therapy, including amiodarone. Once the diagnosis is established, an ICD is recommended for patients who have had ≥1 episodes of hemodynamically unstable, sustained VT or ventricular fibrillation (VF) (class I), those with severe systolic dysfunction of the RV, LV, or both, regardless of the presence of arrhythmias (class I), or those who have had ≥1 episodes of hemodynamically stable, sustained VT (class IIa). Heart transplantation is recommended as a final therapeutic option in ACM patients with either severe, unresponsive congestive heart failure or recurrent episodes of VT/VF that are refractory to catheter (and surgical) ablation in experienced centers and/or ICD therapy [16]. Given the challenging prognosis of ARVC, gene therapy has become an exciting new approach to treatment for ACM. With gene therapy, genes in cells are changed, requiring carriers to deliver genetic material into different cells, tissues, and organs [17].

## Conclusions

Although ACM remains a challenging disease to treat, advances in diagnostic tools and therapeutic options have significantly improved patient outcomes. ICD implantation is crucial for high-risk patients based on guideline-directed indications. Ongoing research into the genetic and molecular mechanisms underlying ACM is crucial for further refining treatment strategies and identifying potential new therapies. With appropriate management, including lifestyle modifications and medical interventions, many ACM patients can lead relatively normal lives, though lifelong monitoring and care are necessary.

## References

[1] Corrado D, Basso C, Judge DP (2017). Arrhythmogenic cardiomyopathy. Circ Res.

[2] Marcus FI, McKenna WJ, Sherrill D (2010). Diagnosis of arrhythmogenic right ventricular cardiomyopathy/dysplasia: proposed modification of the Task Force Criteria. Eur Heart J.

[3] Corrado D, van Tintelen PJ, McKenna WJ (2020). Arrhythmogenic right ventricular cardiomyopathy: evaluation of the current diagnostic criteria and differential diagnosis. Eur Heart J.

[4] Corrado D, Perazzolo Marra M, Zorzi A (2020). Diagnosis of arrhythmogenic cardiomyopathy: the Padua criteria. Int J Cardiol.

[5] te Riele AS, James CA, Groeneweg JA (2016). Approach to family screening in arrhythmogenic right ventricular dysplasia/cardiomyopathy. Eur Heart J.

[6] Ohno S (2016). The genetic background of arrhythmogenic right ventricular cardiomyopathy. J Arrhythm.

[7] Krahn AD, Wilde AA, Calkins H, La Gerche A, Cadrin-Tourigny J, Roberts JD, Han HC (2022). Arrhythmogenic right ventricular cardiomyopathy. JACC Clin Electrophysiol.

[8] Olcum M, Rouhi L, Fan S (2023). PANoptosis is a prominent feature of desmoplakin cardiomyopathy. J Cardiovasc Aging.

[9] Basso C, Corrado D, Thiene G (1999). Cardiovascular causes of sudden death in young individuals including athletes. Cardiol Rev.

[10] Graziano F, Zorzi A, Ungaro S (2024). The 2023 European Task Force criteria for diagnosis of arrhythmogenic cardiomyopathy: historical background and review of main changes. Rev Cardiovasc Med.

[11] Corrado D, Anastasakis A, Basso C (2024). Proposed diagnostic criteria for arrhythmogenic cardiomyopathy: European Task Force consensus report. Int J Cardiol.

[12] Tadros HJ, Miyake CY, Kearney DL (2023). The many faces of arrhythmogenic cardiomyopathy: an overview. Appl Clin Genet.

[13] Roux-Buisson N, Gandjbakhch E, Donal E (2014). Prevalence and significance of rare RYR2 variants in arrhythmogenic right ventricular cardiomyopathy/dysplasia: results of a systematic screening. Heart Rhythm.

[14] James CA, Bhonsale A, Tichnell C (2013). Exercise increases age-related penetrance and arrhythmic risk in arrhythmogenic right ventricular dysplasia/cardiomyopathy-associated desmosomal mutation carriers. J Am Coll Cardiol.

[15] Basso C, Ronco F, Marcus F (2008). Quantitative assessment of endomyocardial biopsy in arrhythmogenic right ventricular cardiomyopathy/dysplasia: an in vitro validation of diagnostic criteria. Eur Heart J.

[16] Corrado D, Wichter T, Link MS (2015). Treatment of arrhythmogenic right ventricular cardiomyopathy/dysplasia: an international task force consensus statement. Eur Heart J.

[17] Ravindran D, Kok C, Farraha M (2020). Gene and cell therapy for cardiac arrhythmias. Clin Ther.

